# Short-axis versus long-axis ultrasound-guided thyroid nodule biopsy: A randomized controlled trial of diagnostic performance in Iraq

**DOI:** 10.1016/j.sopen.2025.11.003

**Published:** 2025-11-14

**Authors:** Mustafa Adnan Zaidan, Hussein Ali Tawfeeq, Ali Kamal Ghanim

**Affiliations:** a Department of Anesthesia Techniques, Al-Farahidi University, Baghdad, 10021, Iraq; b Al-Karkh Health Directorate, Ministry of Health, Baghdad, Iraq

**Keywords:** Thyroid nodule, Ultrasonic-guided fine needle aspiration, Short axis technique, Long axis technique, Diagnostic accuracy, Iraq

## Abstract

**Background:**

Ultrasound guided fine needle aspiration cytology (US-FNAC) is the gold standard of evaluation of thyroid nodule. Two main approaches are available - short axis (perpendicular) and long axis (parallel), and each has theoretical clinical advantages. Evidence comparing the diagnostic performance of the two in Middle Eastern populations is limited. Objective: To compare the sensitivity, specificity, and accuracy of US-FNAC of short-axis versus long-axis in thyroid nodules with implications for patient care.

**Methods:**

A prospective multi-center randomized controlled trial was performed in Pioneer Private Laboratory and Mustafa Hafez Specialized Laboratory, Baghdad, Iraq from March to December 2024. A total of 196 nodules from patients ≥18 years were randomized to undergo short-axis or long-axis US-FNAC. Cytological results by the Bethesda System were correlated with histopathology. Sensitivity, specificity, accuracy, positive predictive value (PPV) and negative predictive value (NPV) were the primary outcomes.

**Results:**

Of 196 nodules (mean age 47.8 ± 13.5 years; 79.6 % female), patient and nodule characteristics did not differ between groups. Long-axis US-FNAC was 73.9 % sensitive, 100 % specific, 87.0 % accurate, 100 % positive predictive and 79.3 % negative predictive. Short-axis US-FNAC showed sensitivity of 76.7 %, specificity of 100 %, accuracy of 89.2 %, positive predictive value of 100 % and negative predictive value of 83.3 %. There were no differences in accuracy of diagnosis (*p* = 0.524).

**Conclusions:**

Short-axis and long-axis US-FNAC offer similar diagnostic performance of thyroid nodules in Iraqi patients. Although there were no differences in accuracy between procedures, operator experience, nodule characteristics, and patient factors could be used to choose the technique that best suited the clinical situation, which would allow for flexibility in clinical practice and potential improvements in patient comfort and procedural efficiency.

## Graphical abstract

**Figure:**
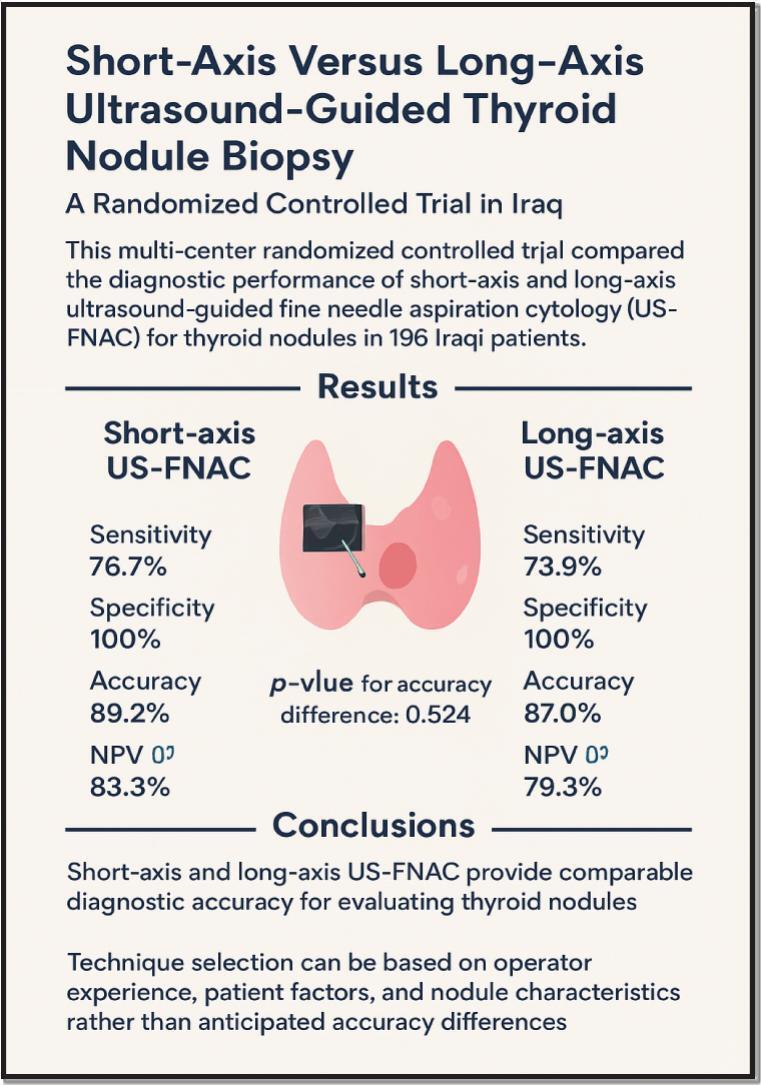

## Introduction

Thyroid nodules are one of the most common endocrine disorders and have a prevalence rate of up to 60 % in the general population based on high resolution ultrasonography [1,2]. Although most thyroid nodules are benign, about 5–15 % of them are malignant, requiring their accurate diagnosis for adequate management [3,4]. Because of the rising incidence of thyroid cancer in the last decade, especially in developing countries, such as the Middle East, this is more important than ever in the development of adequate diagnostic techniques [5].

In Iraq and the Middle East in general, thyroid disorders are a major healthcare burden, and environmental factors, iodine deficiency, and genetic predisposition are factors that cause high prevalence rates [6]. The development of standardized evidence-based diagnostic protocols is essential for optimizing patient outcomes and managing healthcare resources effectively in these healthcare settings.

Ultrasonography has been proven to be the mainstay of imaging modality for evaluation of thyroid nodules, offering a detailed morphological evaluation and guiding the further course of action [7,8]. The combination of ultrasound guiding and fine needle aspiration cytology (US-FNAC) has revolutionised the management of thyroid nodules, with a significant improvement in diagnostic accuracy and lower rates of non-diagnostic sampling compared to palpation guided procedures [9,10].

US-FNAC can be performed with two distinct ways of visualization of the needle: the short-axis (perpendicular) approach, in which the needle can be seen as a hyperechoic dot bisecting the imaging plane, and the long-axis (parallel) approach, in which the whole shaft of the needle is visualized within the ultrasound plane [11,12]. Theoretical advantages of each approach include the long-axis approach, which provides complete visualization of the needle and potentially improved precision, and the short-axis approach, which offers potentially easier manipulation of the needle in difficult anatomic locations, and may be more comfortable for the patient [13].

Despite the widespread adoption of both techniques, previous studies have yielded conflicting results regarding technique superiority one approach over the other. While some investigations have suggested improved specimen adequacy with the long-axis approach [14,15], others have reported comparable outcomes between techniques [16]. Critically, the heterogeneity in study designs, small sample sizes, single-center limitations, operator experience variability, and inconsistent outcome measures has contributed to ongoing uncertainty regarding optimal technique selection.

Most of the existing studies have major methodological limitations: insufficient sample size to draw definitive conclusions, single-center studies limiting generalizability, lack of randomized allocation, limiting sampling bias, inconsistent endpoint definition. Furthermore, no large-scale randomized controlled trials have been conducted in Middle Eastern populations, where genetic, environmental and healthcare system factors may affect performance in diagnosis.

The Iraqi healthcare system that serves a population of more than 40 million and has limited specialized centers needs evidence-based guidelines for optimized diagnostic procedures. The absence of conclusive evidence of a comparison between US-FNAC techniques is a serious knowledge gap that affects clinical decision-making and resources in this region.

### Study objectives

The main aim of this multi-center randomized controlled trial was to provide definitive evidence on the comparison of the diagnostic performance of short axis versus long axis US-FNAC techniques for evaluation of thyroid nodules in Iraqi patients, with special focus on sensitivity, specificity and overall diagnostic accuracy. Secondary objectives were to evaluate nondiagnostic rates, subgroup analysis by nodule characteristics, and evaluate clinical practice implications for technique choice.

## Materials and methods

### Study design and setting

This was a prospective multi-center randomized controlled trial that was also carried out in two specialized centers in Baghdad, Iraq: Pioneer Private Laboratory and Mustafa Hafez Specialized Laboratory. The period of study was 1st March 2024 to 31st December 2024. The two facilities are fitted with high-resolution ultrasounds and experienced cytopathologists and interventional radiologists who have more than 10 years of ultrasound-guided biopsy experience. It was conducted in line with the recommendations of the CONSORT 2010 framework of reporting randomized controlled trials and was approved by the institutional review boards of each of the two centers (IRB approval numbers: PPL-2024-003 and MHSL-2024-007). All participants were informed before enrolling in the study by means of written informed consent.

### Equipment specifications and technical parameters

#### Ultrasound system

Samsung HERA W10 that has the linear array transducers and it is operated at frequencies of 5–12 MHz. Imaging parameters were standardized with depth mode of 4–6 cm, the focal zone was set at nodule level, gain maximized to provide tissue contrast and time gain compensation was optimized to provide equal echogenicity.

#### Needle specifications

All procedures were done with 25-gauge and 1.5-inch disposable needles (BD Medical, Becton Dickinson and Company) and 10 mL disposable plastic syringes.

#### Cytological processing

The specimen was fixed in 95 % ethyl alcohol then standard staining protocol of Papanicolaou. The samples were examined under a microscope with Olympus BX53 microscope and objectives of 10×, 20×, and 40×.

### Participants and sample size

The calculations of sample sizes have been made using expected 10 % difference in diagnostic accuracy of the techniques (long-axis: 85 %, short-axis: 75 %) in addition to power (80 %), level of significance (two-sided, 5 %), and dropout rate (10 %). This generated a required sample size of 98 nodules per group (*n* = 196) using a two proportion compared with proportion formula. The parameters used in the calculation of the statistical power were: accuracy difference expected to be 10 %, alpha error rate of 0.05 (two-sided), beta error rate 0.2 (power = 80 %), dropout rate of 10 %, and 98 patients in each group to make the total sample size 196.

The population of the study included 196 thyroid nodules of consecutive patients who reported to the clinic and requested the evaluation of their thyroid nodules.

**Inclusion criteria were:** (1) thyroid nodules ≥1 cm in maximum diameter, (2) the need to have fine needle aspiration due to the ultrasound appearance by TI-RADS classification, (3) the consent to participate in the study, and (4) the intention to undergo a histopathological correspondence by having a surgical procedure.
**Exclusion criteria were:** (1) pregnancy, (2) bleeding disorders or anticoagulant therapy, (3) inability to provide informed consent, (4) these lesions were all cystic (90 % or more), and (5) fine needle aspiration of the target nodule in the previous six months.

### TI-RADS classification system

Every nodule was categorized by the standardized ACR TI-RADS 2017 system:

**TI-RADS 1:** Normal thyroid gland.
**TI-RADS 2:** Benign nodules (0 % risk of malignancy) - spongiform or partially cystic nodules of suspicious nature.
**TI-RADS 3:** Indeterminate nodules (≤5 % risk of malignancy) - nodules, iso- or hyperechoic solid nodules or partially cystic nodules with eccentric solid nodules, without any suspicious characteristics.
**TI-RADS 4:** Intermediate risk nodules (5–20 % of risk of malignancy) - hypoechoic solid nodules and are not suspicious.
**TI-RADS 5:** Highly suspicious nodules (>20 % risk of malignancy) - solid hypoechoic nodules having one or more suspicious characteristics such irregular margins, micro calcifications, taller than wide shape, rim calcifications with small extrusive soft tissue elements, or extrathyroidal extension.

### Randomization and blinding

Participants who satisfied the eligibility criteria were randomly selected to either short axis or long axis US-FNAC technique through a four permuted block randomization (Random.org). Stratification was done by study center so that there was equal distribution of them among sites. The concealment of allocation was kept by sealed opaque sequentially numbered envelopes. The nature of the intervention could not allow blinding of the performing radiologist. But interpretation of the specimens was done with the cytopathologists being blinded to the method applied, and statistical analysis was done by an investigator blinded on group allocation, until after primary analysis had been completed.

### Patient flow and study progress

**Fig. 1:**
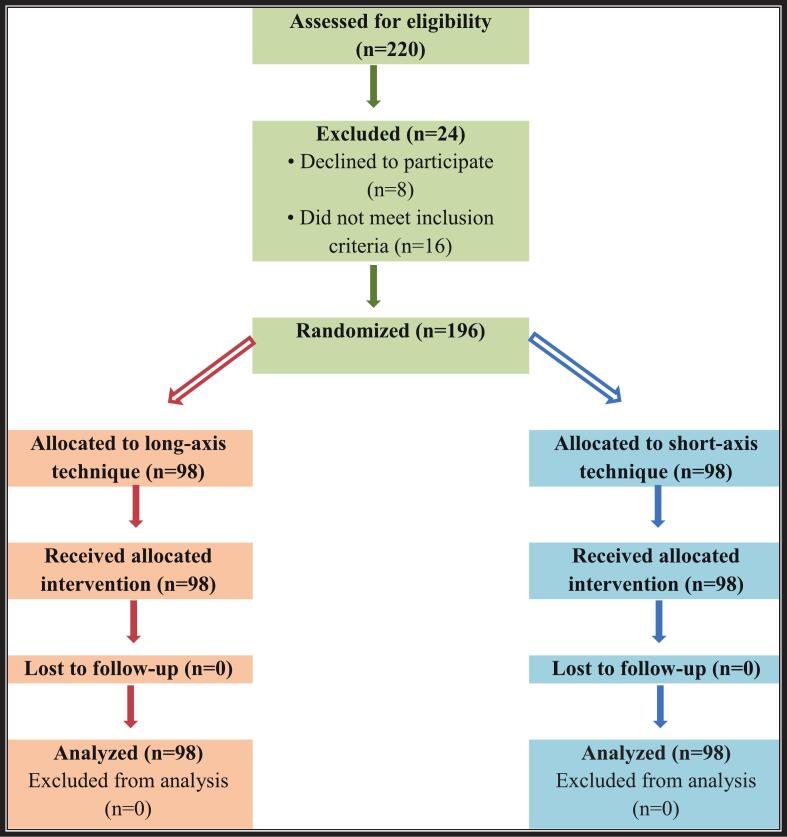
CONSORT flow diagram.

Out of 220 patients, preliminary evaluation was done to determine the eligibility of patients. After including and exclusion criteria were used, there were 196 thyroid nodules in 186 patients included in the ultimate analysis. The overall patient flow was in compliance with the CONSORT guidelines (Fig. 1).

### Quality control and safety monitoring

Extensive quality control was introduced during the research. Standard protocols were trained during pre-study training, which was followed by frequent calibration meetings at the study period. The chosen procedures were filmed to check the technique, so there would be consistency between operators.

Quality assurance was done by having two independent reviews of all specimens, weekly consensus meetings of the discordant cases and as an external quality assurance involvement, and had a blinded re-review of 20 % cases to sustain the accuracy of the diagnosis. The data was managed using electronic case report forms, where range checks were builtin, critical variables were made to have a dual data entry, routine data monitoring and cleaning and audit trails were maintained on all changes made on the database.

Adverse events were identified in safety monitoring procedures as minor (transient pain, minor bleeding <5 min, vasovaginal response) and major (significant hematoma requiring intervention, infection, nerve injury, tumor seeding). Each patient was followed-up with immediate post-operative observation (15 min), telephone follow-up at 24 h, 1-week of clinical follow-up in case of reported complications, and long-term follow-up, which is done by correlation with surgical pathology.

### Intervention protocols

All procedures were performed by two experienced interventional radiologists with >10 years of ultrasound-guided biopsy experience using standardized protocols. A high-frequency linear transducer (5–12 MHz) was employed for all procedures using Samsung HERA W10 ultrasound systems.

**Short-axis technique:** Needle insertion perpendicular to the transducer, with needle tip visualization as a hyperechoic focus. The needle pathway was optimized to avoid critical structures while maintaining perpendicular angulation to the transducer face.
**Long-axis technique:** Needle insertion parallel to the transducer's long axis, allowing complete needle shaft visualization throughout the procedure. The transducer was positioned to optimize needle pathway visualization from skin entry to target lesion.

For both techniques, 25-gauge needles were used with 2–3 passes per nodule. Real-time monitoring ensured optimal needle positioning within the target lesion before aspiration.

### Specimen processing and analysis

Aspirate was instantaneously forced onto glass slides, dried by air and fixed with alcohol to be viewed under the microscope. Papanicolaou staining was done as per the usual protocols. Adequacy of the specimen was based on the 2017 Bethesda System report: the presence of at least six clusters of benign follicular cells (15–20 cells each cluster) or definitive malignancy [17].

The interpretation of the cytological and the Bethesda System of Reporting Thyroid Cytopathology were used with certain categories and management suggestions: (Table 1).

**Table 1:** Bethesda system categories and management recommendations.

Category	Diagnosis	Risk of malignancy	Recommended management
I	Non-diagnostic or unsatisfactory	1–4 %	Repeat FNA with ultrasound guidance
II	Benign	0–3 %	Clinical and sonographic follow-up
III	Atypia of undetermined significance (AUS) or follicular lesion of undetermined significance (FLUS)	10–30 %	Repeat FNA, molecular testing, or lobectomy
IV	Follicular neoplasm or suspicious for follicular neoplasm	25–40 %	Lobectomy or molecular testing
V	Suspicious for malignancy	50–75 %	Near-total thyroidectomy or lobectomy
VI	Malignant	97–99 %	Near-total thyroidectomy

All slides underwent independent review by two experienced cytopathologists, with discordant cases resolved through consensus. Inter-observer agreement was assessed using Cohen's kappa coefficient. Surgical specimens were processed using standard histopathological techniques, with 4-μm sections stained with hematoxylin and eosin for microscopic examination by dedicated endocrine pathologists.

### Statistical analysis

Statistical analysis was done with the SPSS 28.0 version. The statistical tests used were independent samples *t*-test or Mann-Whitney *U* test (depending on the normality of the distribution), chi-square test or Fisher exact test (when the expected frequencies <5), Wilson score method to test the confidence intervals of diagnostic accuracy measures, and Cohen kappa coefficient to test inter-rater agreement. The handling of missing data was done using a complete case analysis method of primary outcomes, and sensitivity analysis (omission of non-diagnostic cases). There was no imputation since there was little missing data (<5 %).

Continuous variables were given in the form of mean ± standard deviation and categorical variables were given in frequencies and percentages. Continuous variable comparisons were done by independent *t*-tests and the same was done with chi-square tests or Fisher exact tests with categorical variables.

Diagnostic performance parameters (sensitivity, specificity, accuracy, PPV, and NPV) were calculated using standard formulas, with 95 % confidence intervals calculated using the Wilson score method. For diagnostic accuracy calculation, Bethesda categories IV-VI were considered positive, while categories II-III were considered negative. Category I (non-diagnostic) specimens were excluded from primary analysis but reported separately.

Stratified subgroup analysis was conducted based on nodule size (<2 cm vs ≥2 cm), TI-RADS, and patient age. All comparisons were regarded as statistically significant at a *p*-value of less than 0.05.

## Results

### Patient characteristics and safety outcomes

The age of the patients was between 19 years and 78 years (mean age of 47.8 ± 13.5 years), where 156 (79.6 %) female respondents were included. There was no difference in the number of nodules produced in each method where 98 nodules were in each group. Monitoring of safety showed high procedures tolerance with the total procedures of 196. Minor complications were found in 12 patients (6.1 %), which were equally divided into long-axis and short-axis groups 6 (6.1 %) and 6 (6.1 %), respectively. There were no significant complications (0 %). The opposing difference between groups was not statistically significant (*p* = 1.000). There were no statistically significant variations in terms of age (*p* = 0.412), gender distribution (p = 1.000), nodule laterality (*p* = 0.615), nodule size (*p* = 0.587), or the ultrasound features such as the TI-RADS classification (*p* = 0.184) (Table 2).

**Table 2:** Baseline characteristics according to FNA technique.

Variable	Long-axis (*n* = 98)	Short-axis (n = 98)	P-value
Age (years), mean ± SD	46.8 ± 12.9	48.7 ± 14.1	0.412
Female gender, n (%)	78 (79.6)	78 (79.6)	1.000
Right-sided nodule, n (%)	52 (53.1)	49 (50.0)	0.615
Nodule size (cm), mean ± SD	2.4 ± 1.2	2.3 ± 1.1	0.587
Bethesda category			0.892
I (non-diagnostic)	6 (6.1)	5 (5.1)	
II (benign)	38 (38.8)	42 (42.9)	
III (AUS/FLUS)	18 (18.4)	16 (16.3)	
IV (FN/SFN)	12 (12.2)	13 (13.3)	
V (suspicious)	14 (14.3)	12 (12.2)	
VI (malignant)	10 (10.2)	10 (10.2)	
TI-RADS classification			0.184
TI-RADS 2	8 (8.2)	5 (5.1)	
TI-RADS 3	26 (26.5)	33 (33.7)	
TI-RADS 4	52 (53.1)	48 (49.0)	
TI-RADS 5	12 (12.2)	12 (12.2)	

### Diagnostic performance analysis

Surgical correlation was done and histopathological analysis showed that 89 (45.4 %) cases were malignant. Malignant cases were distributed similarly between techniques; 46 (47.9 %) and 43 (44.8 %) in the long-axis and short-axis groups respectively (*p* = 0.652) (Table 3).

**Table 3:** Comparative diagnostic performance of short-axis and long-axis techniques.

Technique	Bethesda category	Malignant	Benign	Total^a^	Diagnostic parameters
Long-axis	Positive (IV–VI)	34	0	34	Sensitivity: 73.9 % (95 % CI: 59.7–85.2 %) Specificity: 100 % (95 % CI: 92.3–100 %) Accuracy: 87.0 % (95 % CI: 78.8–92.9 %) PPV: 100 % (95 % CI: 89.7–100 %) NPV: 79.3 % (95 % CI: 67.2–88.5 %)
	Negative (II–III)	12	46	58	
	Total	46	46	92	
Short-axis	Positive (IV–VI)	33	0	33	Sensitivity: 76.7 % (95 % CI: 62.0–87.7 %) Specificity: 100 % (95 % CI: 92.9–100 %) Accuracy: 89.2 % (95 % CI: 81.5–94.5 %) PPV: 100 % (95 % CI: 89.4–100 %) NPV: 83.3 % (95 % CI: 71.5–91.7 %)
	Negative (II–III)	10	50	60	
	Total	43	50	93	

a Excluding non-diagnostic cases (6 in the long-axis group and 5 in the short-axis group).

#### Primary outcome comparison

The difference between the techniques in the diagnostic accuracy was not statistically significant (87.0 % vs 89.2 %, p = 0.524). In the same manner, there were no noteworthy variations in sensitivity (*p* = 0.743), specificity (*p* = 1.000), PPV (p = 1.000), or NPV (*p* = 0.569).

##### Secondary outcomes

The non-diagnostic rates were also low in both groups: 6.1 (95 % CI: 2.3–12.8 %) with long-axis and 5.1 (95 % CI: 1.7–11.5 %) with short-axis methods (*p* = 0.751). The inter-observer agreement on cytological interpretation was excellent (k = 0.89, 95 % CI = 0.83–0.95).

##### Subgroup analysis

The nodule size and TI-RADS category of the subgroup analysis showed that the long-axis and short-axis techniques had similar accuracy in diagnosis. With nodules <2 cm, the short-axis method was marginally more accurate (87.9 % vs. 85.7 %) and for nodules ≥2 cm, the short-axis technique again was marginally higher accurate (90.5 % vs. 88.2 %) though not significantly different (*p* > 0.05). Similarly, TI-RADS stratification also found almost identical results with the two methods in nodules of higher risk (TI-RADS 5, 91.7 % of both) and a slight superiority in short-axis accuracy in TI-RADS 3–4 (88.7 % vs. 86.5 %), but with no statistical significance (Table 4).

**Table 4:** Diagnostic Accuracy by Nodule Size and TI-RADS Category.

Subgroup	Nodule/category	Long-axis accuracy	Short-axis accuracy	p-Value
Nodule size	<2 cm	85.7 %	87.9 %	0.746
Nodule size	≥2 cm	88.2 %	90.5 %	0.682
TI-RADS	3–4	86.5 %	88.7 %	0.614
TI-RADS	5	91.7 %	91.7 %	1.000

Note: No statistically significant differences were observed in any subgroup analysis (all p > 0.05).

## Discussion

### Interpretation of clinical significance and diagnostic accuracy

Accurate thyroid nodule diagnosis through US-FNAC is essential for preventing unnecessary thyroidectomy and associated complications, reducing healthcare burden, and optimizing patient outcomes (PMID: 40057484). Sensitivity of 73.9–76.7 % shows that most of the malignant nodules are being correctly diagnosed and specificity of 100 % is that benign nodules are being separated safely and reliably on the nodules that are found to be malignant. This high specificity is particularly clinically significant as it minimizes false-positive diagnoses, preventing unnecessary surgical interventions and their associated morbidity and healthcare costs.

This multi-centered randomized controlled trial is currently the most suitable evidence on the comparison between short and long axis ultrasound-guided fine needle aspiration methods of assessing thyroid nodules. We have shown that both methods possess a high level of diagnostic accuracy without any statistically significant differences between different primary and secondary outcome measures.

The diagnostic performance parameters found in our study are consistent with the systematic reviews and meta-analyses. The overall accuracy of both methods (87.0–89.2 %) is in the anticipated range of US-FNAC procedures conducted by skilled operators, and implies the external validity of our results [18,19].

The findings we obtained are quite different compared to the initial research by Kandil et al. [20] who listed significant differences in the diagnostic value of the methods (93.9 % vs 61.3 %). This difference is probably due to a number of methodological considerations: they have fewer patients (*n* = 50), a single center design, they could be biased by the operators, and the patient population is different. The more reliable evidence is given by our bigger and multi-centered design with standardized protocols and experienced operators.

The recent research has given diverse results; some found benefits of the long-axis method [21,22] and others reported similar results [23,24]. The variation in the research findings is probably because of the differences in:

o **Operator Experience:** The advantage of our study is that radiologists with high experience (>10 years) participated, and it could be the reason behind the high performance of the two techniques. The long-axis approach which provides full visualization of the needle might be of a benefit to less experienced operators.
o **Patient Population:** Our Middle Eastern population could possess a variation of nodules and there could be genetic and environmental differences that could affect nodule morphology and diagnostic difficulties than the Western population.
o **Technical Standardization:** The application of the same ultrasound equipment, standardized protocols and strict quality control measures were probably the reason behind similar results of the techniques.
o **Sample Preparation and Interpretation:** The application of immediate specimen adequacy testing and professional cytopathological examination reduced the quality of the specimen due to the use of techniques.

### Clinical practice implications

This similarity in the diagnostic performance of techniques has significant clinical practice implications:

#### Technique selection strategy

The strategy of choosing techniques is not to follow a one-size-fits-all technique but practice can choose techniques depending on the level of operator comfort and experience, patient anatomy, position limits, nodule location and accessibility, patient comfort and anxiety, and training and institutional bias.

#### Implications of training

Medical education programs and continuing medical education programs can be developed to master either technique instead of both and this could result in greater competency in general and decrease the complexity of training.

#### Resource optimization

Both methods could be used by the healthcare systems and rely on current expertise and training ability without any fear of affected diagnostic accuracy, which makes the process of resource distribution more efficient.

#### Patient-centered care

The choice of technique is flexible and can be optimized according to patient-specific factors, which might lead to an increase in patient comfort and tolerance of the procedures.

### Regional healthcare context

To the Iraqi healthcare system and the Middle East in general, these findings indicate the use of standardization opportunities, where both methods can be applied safely at different levels of healthcare without diagnostic quality being compromised, training efficiency where the limited specialist training resources can be used to achieve excellence in either of the two techniques as opposed to requiring dual competency, and quality assurance, where with the right training and quality control measures, excellent results can be achieved in resource-limited environments.

### Study strengths

o First large-scale randomized controlled trial comparing US-FNAC techniques in the Middle East
o Multi-center design enhancing generalizability
o Rigorous randomization and allocation concealment
o Standardized protocols and experienced operators
o Comprehensive statistical analysis with appropriate power calculation
o Blinded cytopathological interpretation
o Complete histopathological correlation for all cases

### Study limitations

o This may represent a best-case scenario, and the performance of each technique might vary more significantly among trainees or less experienced operators.
o Relatively short follow-up period may not capture long-term outcomes or rare complications
o Cost-effectiveness analysis was not performed, which may influence technique selection in resource-limited settings
o Single geographic region may limit global generalizability
o Potential learning curve effects not assessed for operators transitioning between techniques

### Future research directions

Several research avenues warrant investigation: experience-stratified studies comparing technique performance across different operator experience levels to inform training requirements, cost-effectiveness analysis including procedure time, complication rates, and resource utilization to guide policy decisions, artificial intelligence integration investigating AI-assisted needle guidance systems that may optimize both techniques, patient-reported outcomes assessment of patient comfort, anxiety, and preference between techniques to inform patient-centered care approaches, and long-term follow-up studies to assess technique-specific complication rates and diagnostic stability over time.

## Conclusions

In this multi-center randomized controlled trial, the short-axis and the long-axis ultrasound-guided fine needle aspiration methods have shown similar diagnostic potentials in evaluating thyroid nodules in patients of Iraq. Operators experience, patient-specific and nodule characteristics should dictate the decision on the choice of techniques instead of the expected differences in diagnostic ideals. These two methods are equally appropriate in the evaluation of thyroid nodules in the hands of trained experts as long as they apply standard guidelines. The evidence confirms the possibility of flexible technique in clinical practice, which can benefit the patient, the efficiency of the procedure, and the optimization of resources and does not compromise the quality of diagnostics. To the healthcare systems in Iraq and other such environment, the findings can be seen as a motivational factor that good diagnostic results can be obtained through both methods and by training and implementing the best method in that particular country based on the available knowledge and available resources instead of worrying about superiority in the diagnosis.

## CRediT authorship contribution statement

**Mustafa Adnan Zaidan:** Writing – original draft, Investigation, Data curation, Conceptualization. **Hussein Ali Tawfeeq:** Writing – review & editing, Methodology, Formal analysis, Conceptualization. **Ali Kamal Ghanim:** Writing – review & editing, Supervision, Investigation, Conceptualization.

## Ethical approval statement

The study was approved by the institutional ethics committees of Pioneer Private Laboratory (IRB approval number: PPL-2024-003) and Mustafa Hafez Specialized Laboratory (IRB approval number: MHSL-2024-007), Baghdad, Iraq. Written informed consent was obtained from all participants.

## Funding source

This research did not receive any specific grant from funding agencies in the public, commercial, or not-for-profit sectors.

## Declaration of competing interest

The authors declare no conflicts of interest.
